# Imaging predictors of successful surgical treatment of hemifacial spasm

**DOI:** 10.1093/braincomms/fcab146

**Published:** 2021-08-06

**Authors:** Marion A Hughes, Katie S Traylor, Barton F Branstetter IV, Komal P Eubanks, Yue-Fang Chang, Raymond F Sekula Jr

**Affiliations:** 1 Department of Radiology, University of Pittsburgh Medical Center, Pittsburgh, PA 15213, USA; 2 Department of Otolaryngology, University of Pittsburgh Medical Center, Pittsburgh, PA 15213, USA; 3 Department of Neurosurgery, University of Pittsburgh Medical Center, Pittsburgh, PA 15213, USA

**Keywords:** cranial nerves: decompression neurosurgery, neuroanatomy, movement disorder: imaging, cranial neuralgia

## Abstract

Identify preoperative imaging findings in hemifacial spasm patients that predict the post-surgical success following microvascular decompression. This is a retrospective study of patients who were diagnosed with hemifacial spasm, had a dedicated cranial nerve MRI, and underwent microvascular decompression for hemifacial spasm. Bilateral facial nerves were interrogated for neurovascular compression. If neurovascular compression was identified, we recorded whether the offending vessel was an artery, a vein or both. The location of the neurovascular compression (proximal nerve versus distal nerve) was noted. The severity of the neurovascular compression was categorized as contact versus deformity of the nerve. Patients were contacted to determine their post-operative spasm status. The relationships between imaging findings and post-surgical outcome were assessed by Chi-square tests, and odds ratios were calculated to quantify the degree of association. The study included 212 patients. Upon follow up, 192 patients were spasm free (90.57%). Imaging findings on the symptomatic side were as follows: arterial neurovascular compression was seen in 207 patients (97.64%), venous only neurovascular compression in two patients (0.94%), and no neurovascular compression in three patients (1.42%). Arterial neurovascular compression along the proximal, susceptible segment of the nerve was observed in 202 patients (95.28%); deformity was observed more commonly than contact alone. Arterial neurovascular compression along the distal segment only of the nerve was observed in five patients (2.36%). In patients with arterial neurovascular compression of the proximal and distal portions of the nerve, 93.07% and 60.0% of patients were spasm-free respectively. If venous neurovascular compression only was observed on imaging, 0% of patients were spasm-free. Patients with arterial neurovascular compression of the susceptible segment are much more likely to be spasm free than patients without this imaging finding, [odds ratio 20.14 (CI 5.08, 79.81), *P*-value <0.0001]. When comparing the two groups of arterial neurovascular compression (deformity versus contact), no statistically significant difference in outcomes was observed. In patients with hemifacial spasm undergoing microvascular decompression, imaging findings do predict surgical outcome. Patients with arterial neurovascular compression of the proximal, susceptible portion of the nerve are much more likely to be spasm free after surgery than those without this imaging finding. The imaging findings inform the risk benefit analysis and discussion with patients before they undergo microvascular decompression for hemifacial spasm.

## Teaser

Hughes et al. found that preoperative magnetic resonance imaging findings predict surgical outcome in patients with hemifacial spasm undergoing surgical decompression of the facial nerve. Patients with arterial neurovascular compression of the proximal, susceptible portion of the nerve are much more likely to be spasm-free than those without this imaging finding.

## Graphical Abstract

**Graphical Abstract:**
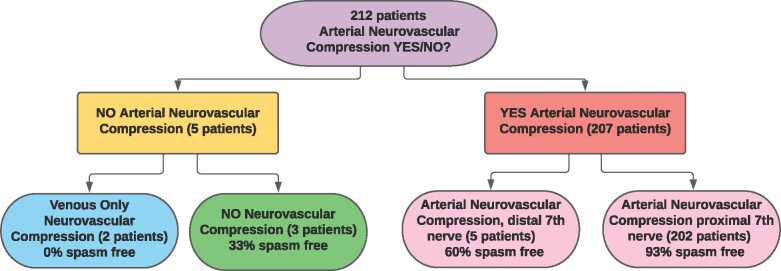

## Introduction

Hemifacial spasm (HFS) is a debilitating neuromuscular movement disorder characterized by highly synchronized, involuntary contractions of the facial muscles. Neurovascular compression (NVC) of the centrally myelinated portion of the facial nerve [i.e. from the root exit point (RExP) of the nerve near the pontomedullary sulcus to just beyond its detachment from the pons] may result in HFS.^1–3^ Botulinum toxin injections^4^ and microvascular decompression (MVD) surgery are the only treatment options for patients with HFS.^5–7^ MVD is a procedure that addresses the propose; lrēd pathogenesis of HFS by relieving NVC along the centrally myelinated portion of the facial nerve, completely relieving spasms in a large majority of patients with a low incidence of morbidity.^8^

A recent MRI study of patients meeting the clinical and electrophysiological of criteria of HFS demonstrated that severe arterial NVC along the susceptible portion of the facial nerve is more common on the symptomatic rather than asymptomatic sides.^9^ With the assumption that characteristics of NVC may be important in understanding the aetiology of HFS and selection of patients for surgery, as it is with trigeminal neuralgia,^7^^,^^10–13^ the aim of this study was to identify preoperative imaging predictors of surgical success in patients with HFS undergoing MVD.

## Materials and methods

This was a retrospective study of patients who underwent MVD for HFS at the (blinded) Hospital. All patients who underwent MVD for HFS at our institution were evaluated and diagnosed with HFS by a single neurosurgeon, who specializes in cranial nerve disorders.

The study population initially consisted of all patients who were diagnosed with HFS, had a dedicated cranial nerve 3 Tesla (T) MRI at our institution, underwent MVD for HFS at (blinded) hospital between 22 April 2014 and 14 August 2019, and had at least 1 year of post-surgical follow-up. Patients were excluded if they had a prior history of MVD for HFS on the symptomatic side, less than 1 year of follow-up, did not undergo a dedicated cranial nerve 3 T MRI at our institution, if the MRI was excessively degraded by artefact, or if the patient could not be reached to determine the surgical outcome despite three attempts. This study was HIPAA compliant, and IRB approved.

### MR imaging technique

The MR examinations were performed on a 3 T MR scanner (GE Healthcare, Milwaukee, WI). The MRIs were performed using our cranial nerve protocol, which has been previously published.^3^ In brief, the protocol consists of sagittal T_1_, axial Fluid-Attenuated Inversion Recovery and Diffusion Weighted Imaging sequences of the whole brain with additional multiplanar thin-slice Steady-State Free Precession (SSFP) images through the cranial nerves.

### Anatomic terms

**Figure 1:**
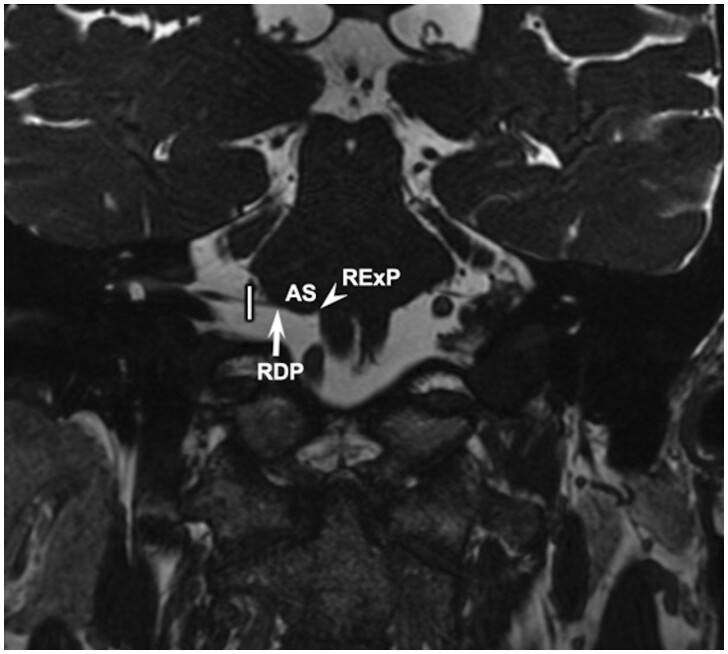
**Anatomy.** Coronal SSFP image demonstrates the locations of the root exit point (RExP, white arrowhead), the attached segment (AS) along the ventral pons, and the root detachment point (RDP, white arrow) of the facial nerve. The proximal cisternal segment extends approximately 3 mm distal to the RDP. The susceptible portion of the facial nerve extends from the RExP (white arrowhead) to the lateral margin of the cisternal segment (white line).

**Figure 2:**
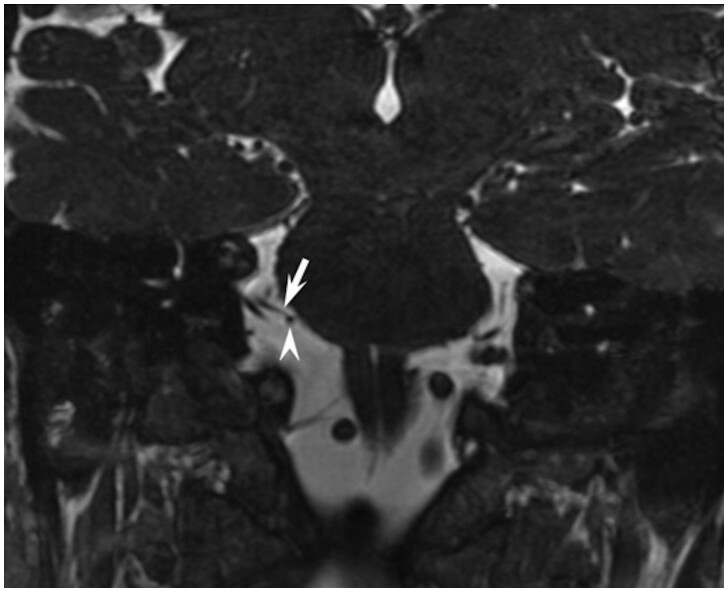
**Neurovascular contact in the proximal cisternal segment.** Coronal SSFP image demonstrating the anterior inferior cerebellar artery (white arrowhead) contacting the proximal cisternal segment of the facial nerve (white arrow) without corresponding displacement of the nerve.

**Figure 3:**
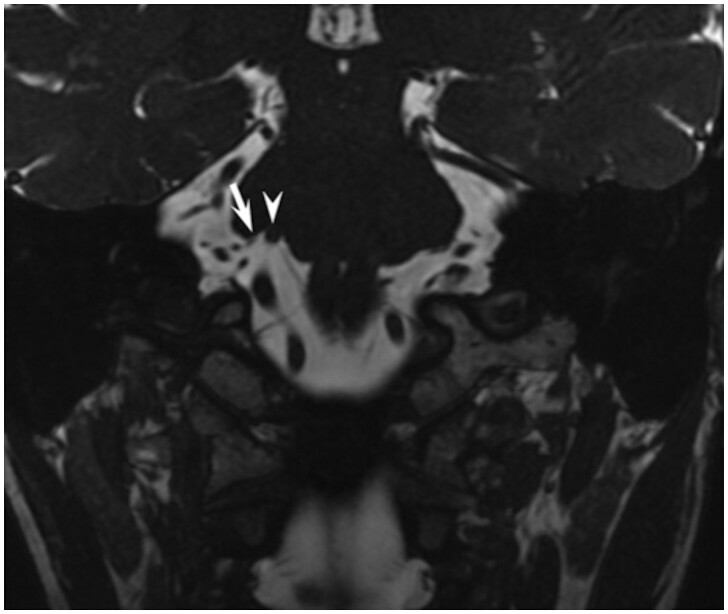
**Neurovascular deformity in the attached segment.** Coronal SSFP demonstrating the vertebral artery (white arrowhead) deforming the ventral surface of the pons along the expected course of the attached segment of the facial nerve. Also shown is the root detachment point of the facial nerve (white arrow).

We adapted anatomic terms utilized by Tomii et al.^14^ and Campos-Benitez et al.^1^ The facial nerve exits the brainstem near the pontomedullary sulcus, termed the RExP. The attached segment of the facial nerve extends from the RExP until the nerve detaches from the pons, referred to as the root detachment point (RDP). Between the RExP and RDP, the facial nerve is tightly bound to the ventral surface of the pons for approximately 8–10 mm, termed the attached segment.^1^^,^^3^^,^^14^ The proximal cisternal segment of the facial nerve extends from the RDP to the area of transition from central to peripheral myelin, which occurs between 1- to 3-mm lateral to the RDP. We define the proximal cisternal segment as the 3 mm segment lateral or distal to the RDP.^3^^,^^14^ The distal cisternal segment of the facial nerve extends from the lateral margin of the proximal cisternal segment to the porus acusticus (Fig. 1). The canalicular segment extends from the porus acusticus to the internal auditory canal fundus. The susceptible portion of the nerve is considered any part of the facial nerve from the RExP in the ponto-medullary sulcus to the lateral margin of the proximal cisternal segment (Fig. 1).^3^^,^^14^ The resistant or non-susceptible portion of the nerve is any part of the nerve distal to the proximal cisternal segment. NVC is defined as any point where a vessel touches the intracranial facial nerve. The severity of the NVC was graded as either contact (Fig. 2) or deformity (Fig. 3), with deformity being the more severe form of NVC. Contact alone is where there was no discernable cerebrospinal fluid between the vessel and the facial nerve but no displacement of the facial nerve at the site of contact. If the vessel displaces the facial nerve or indents the ventral pons along the course of the attached segment, this is considered deformity.

### Image interpretation

Blinded to the symptomatic side, a neuroradiologist (KST) evaluated both facial nerves for NVC along the proximal susceptible and more distal non-susceptible portions of the facial nerve. If NVC was identified, it was noted whether the contacting vessel was an artery, a vein or both. The site of NVC was categorized as occurring along the attached segment along the ventral pons (extending from the RExP to the RDP inclusive), proximal cisternal segment (3 mm lateral to the RDP) or distal segment (distal cisternal and/or canalicular segments) of the facial nerve. If the NVC occurred along the attached segment or proximal cisternal segment, this was considered NVC along the susceptible portion of the nerve. Finally, the severity of the NVC was categorized as contact versus deformity of the nerve. In cases where both contact and deformity occurred on the same nerve, it was categorized as deformity as this is the more severe of the two forms of NVC. Similarly, if NVC occurred along both the susceptible and distal segments, this was recorded as NVC along the susceptible segment.

Blinded to the symptomatic side and surgical results, neuroradiologists (KST, MAH), reviewed the first 20 patients’ MRIs, and kappa coefficients were calculated to determine the inter-rater reliability. Kappa coefficients were determined for three criteria: presence of any arterial contact, site of contact along the facial nerve and severity of NVC.

### Operative technique

MVD of the facial nerve was performed as previously described.^15^ All operations were performed by a single neurosurgeon (RS). During exposure of the facial nerve, the facial nerve was exposed from the pontomedullary sulcus (i.e. RExP) along the attached segment of the facial nerve on the ventral pons, and a few millimetres past the nerve’s RDP from the pons.

### Clinical follow-up

All patients were contacted by telephone by either the surgeon (RS) or nurse practitioner (KE) to determine their post-operative spasm status, which was categorized as spasm free, ≥75% improvement, <75% but ≥50% improvement, <50% improvement. Three attempts were made to contact each patient. Contact method attempts included phone, email and traditional mail.

### Statistical analysis

Continuous measures were presented as mean (SD) and categorical measures as *n* (%). The Kappa statistics were estimated to assess the inter-rater reliability in the initial double-read examinations. The relations of imaging findings and post-surgical outcome were assessed by Chi-square tests and odds ratios were calculated to quantify the degree of association. Odds ratios (ORs) are reported OR [0.95 confidence interval (CI)]. All tests were two-tailed with an alpha set to 0.05. Analyses were conducted using SAS v9.4 (SAS Institute, Cary, NC).

### Data availability

The authors confirm that the data supporting the findings of this study are available within the article.

## Results

There were 212 patients who met inclusion criteria, 152 (71.7%) female patients and 60 (28.3%) male patients. Symptoms were right sided in 95 patients (44.81%) and left sided in 117 (55.19%). The average age of the patient at the time of MVD was 56.22 years, SD 11.32 years, range 20.0–88.0 years (Table 1). The mean follow-up interval was 3.97 years, SD 1.32 years, range 1.77–6.31 years. The median follow-up interval was 3.97 years. A total of 30 patients were excluded [prior MVD (20 patients), MRI degraded by excessive motion (1 patient), unable to contact despite three attempts (9 patients)]. All imaging findings analysed and presented are for the symptomatic nerve. We have previously reported the imaging findings comparing symptomatic versus asymptomatic nerves in patients with HFS.^9^

**Table 1:** Patient characteristics and overall group outcomes

HFS side	Right	Left		
*N* (%)	95 (44.81%)	117 (55.19%)		
**Gender**	**Male**	**Female**		
*N* (%)	60 (28.3%)	152 (71.7%)		
**Age, years**	**Mean**	**SD**	**Median**	**range**
	56.22	11.32	57	20.0–88.0
**Spasm outcome**	**Spasm free**	**≥75% improvement**	**≥50% but** < **75% improvement**	**<50% improvement**
	192 (90.57%)	8 (3.77%)	6 (2.83%)	6 (2.83%)

### Inter-rater reliability

For the 25 double-read examinations, kappa coefficients were determined for three variables: presence of any arterial NVC, site of contact along the facial nerve and severity of NVC. All three criteria had substantial interrater reliability with Kappa values ranging from 0.75 to 1. The kappa coefficient for any arterial NVC versus no arterial NVC was 1 (0.95 CI = 1–1), for location of NVC along the facial nerve the kappa coefficient was 0.78 (0.95 CI = 0.37–1), for severity of NVC and a kappa coefficient of 0.75 (0.95 CI = 0.42–1).

### Patient outcomes following MVD

Upon follow up, 192 patients were spasm free (90.57%, 192/212), 8 patients (3.77%, 8/212) had ≥75% improvement, 6 patients (2.83%, 6/212) had ≥50% but <75% improvement, and 6 patients (2.83%, 6/212) had <50% improvement (Table 1).

### Vessel type

NVC (artery, vein or both) was seen in 209 patients (98.58%, 209/212). Arterial NVC (arterial NVC alone or arterial and venous NVC) was seen in 207 patients (97.64%, 207/212). Of the patients with arterial NVC, arterial NVC alone (198 patients) was much more common than arterial and venous NVC (9 patients). Venous only NVC was observed in 2 patients (0.94%, 2/212). No NVC was observed in 3 patients (1.42%, 3/212).

### Location of NVC

Arterial NVC along the susceptible segment of the nerve was observed in 202 patients (95.28%, 202/212). Arterial NVC along the distal segment only (no NVC along the susceptible segment) of the nerve was observed in 5 patients (2.36%, 5/212).

### Grade of NVC

Deformity was observed more commonly than contact alone. Specifically, among the 202 patients with arterial NVC along the susceptible portion of the nerve, 57 patients (28.21%, 57/202) exhibited contact and 145 patients (71.78%, 145/202) exhibited deformity.

### Patient outcomes by imaging category

Overall, 93.07% (188/202) of patients with arterial NVC of the susceptible portion of the nerve were spasm free following MVD. Of the patients with arterial NVC of the susceptible portion with deformity (i.e. the most severe grade of NVC), 93.79% (136/145) of patients were spasm-free post-MVD at last follow-up. Of the patients with arterial NVC of the susceptible portion with contact (i.e. less severe grade of NVC), 91.23% (52/57) of patients were spasm-free post-MVD at last follow-up. In patients with arterial NVC of the distal (non-susceptible) portion of the nerve, 60.00% (3/5) of patients were spasm-free at last follow-up. If venous NVC only was observed on imaging, 0% (0/2) of patients were spasm-free at last follow-up. In the three patients with no NVC observed on imaging, only 1 patient (33.33%, 1/3) was spasm-free on follow-up. The above imaging results are summarized in Table 2.

**Table 2:** Imaging findings and percentage of patients spasm free

	*N*	% total	*N* spasm free	% spasm free
Any NVC	209	98.58%	192	90.57%
Vessel type				
Artery				
Artery alone	198	93.40%	184	92.93%
Artery and vein	9	4.25%	7	77.78%
Vein only	2	0.94%	0	0.00%
No NVC	3	1.42%	1	33.33%
Location				
Arterial NVC susceptible	202	95.28%	188	93.07%
Arterial NVC distal only	5	2.36%	3	60.00%
Grade				
Arterial NVC susceptible				
Deformity	145	68.40%	136	93.79%
Arterial NVC susceptible				
Contact	57	26.89%	52	91.23%

For each key imaging finding group, the odds ratio of being spasm free comparing patients with and without this imaging finding are presented in Table 3. Importantly when comparing the group of patients with arterial NVC of the susceptible segment to the group without (venous NVC, distal segment arterial NVC or no NVC), patients with this finding were approximately 20 times more likely to be spasm free [odds ratio 20.14 (CI 5.08, 79.81), *P*-value <0.0001]. When patients with arterial NVC of the susceptible portion (contact only) were compared to the group with no arterial NVC, the former group was approximately 42 times more likely to be spasm-free post-MVD [odds ratio 41.6 (CI 3.87, 447.56), *P*-value 0.002]. When patients with arterial NVC of the susceptible portion (deformity) were compared to the group with no arterial NVC, the former group was 60 times more likely to be spasm-free post-MVD [odds ratio 60.44 (CI 6.10, 598.55), *P*-value 0.001]. Finally, when comparing the two groups of arterial NVC of the susceptible portion (deformity versus contact), no statistically significant difference in outcomes was observed. The odds ratio of being spasm free group comparisons are summarized in Table 4.

**Table 3:** Odds ratio of being spasm free categorized by imaging findings

Imaging category	*N*	% total	*N* spasm free	% spasm free	**Odds ratio** ^a^ **(95% CI)**	*P*-value
Any NVC	209	98.58%	191	91.39%	21.22(1.83, 245.59)	0.015
Arterial NVC susceptible segment	202	95.28%	188	93.07%	20.14 (5.08, 79.81)	<0.0001
Arterial NVC distal segment nerve	5	2.38%	3	60.00%	0.14 (0.02, 0.91)	0.040
Venous NVC only	2	0.94%	0	0.00%	0.02 (0.001, 0.42)	0.001

a Odds ratio when groups with and without this imaging finding were compared.

**Table 4:** Odds ratio of being spasm free, group comparison

Imaging category (*n*) Column 1	Imaging category (*n*) Column 2	**Odd ratio of being spasm free** ^a^	*P*-value
Arterial NVC Susceptible segment Contact (57)	Arterial NVC	6.93 (0.93, 51.79)	0.059
	Distal segment (5)		
Arterial NVC Susceptible segment Deformity (145)	Arterial NVC	10.07 (1.49, 68.18)	0.018
	Distal segment (5)		
Arterial NVC Susceptible segment Contact (57)	No Arterial NVC (5)	41.60 (3.87, 447.56)	0.002
Arterial NVC Susceptible segment Deformity (145)	No Arterial NVC (5)	60.44 (6.10, 598.55)	0.001
Arterial NVC Susceptible segment Deformity (145)	Arterial NVC	1.45 (0.47, 4.54)	0.520
	Susceptible segment		
	Contact (57)		

a Odds ratio of being spasm free when column 1 patient group is compared to column 2 patient group.

## Discussion

There are several important results of our study. Following MVD, 90.57% of patients were spasm free at 1 year follow-up. There are imaging findings that predict surgical outcome. Patients with arterial NVC of the susceptible segment are much more likely to be spasm free than patients without this imaging finding. Specifically, when comparing the group of patients with arterial NVC of the susceptible segment to the group without (venous NVC, distal segment arterial NVC or no NVC), patients with this finding were approximately 20 times more likely to be spasm free. Only in patients with arterial NVC of the susceptible segment (either contact of deformity) were the percentage of patients’ spasm free post-MVD above 90% (91.23%, 93.79%, respectively). Finally, while a slightly greater percentage of patients with the more severe grade of NVC (deformity) were spasm free post-MVD, this was not statistically significant.

Interest in visualization of cranial nerves and surrounding anatomic structures by MRI^16^ has gradually increased, particularly with the development and incorporation of heavily T_2_-weighted, SSFP sequences.^17^^,^^18^ Continued refinement in MRI visualization techniques for cranial neuralgias has led to the increased reliance on MRI by clinicians to assist in appropriateness and planning of referable surgery for cranial neuralgias and other disorders within the cerebellopontine angle.^9^^,^^19–21^ This study provides MRI evidence in support of prior intraoperative observations^1^^,^^22–24^ regarding the location of neurovascular compression in patients with HFS. Postmortem^25^ and intraoperative observations^26^^,^^27^ have long suggested that the cause of HFS is associated with vascular compression of the facial nerve, particularly along the pons^28^ in a majority of patients. One challenge for clinicians attempting to understand the utility of MRI in the surgical management of cranial neuralgias has been the persistent (i.e. even in recent literature) use of the terms ‘root entry/exit zones’. Despite the ubiquitous persistence of the terms, ‘root entry/exit zones’ in the literature, these terms are vague, misleading, and should be abandoned. This study supports our prior observations as well those of Teton et al.^29^ regarding the utility of high-resolution MRI^22^^,^^30^ in the preoperative work-up of patients with HFS and further characterizes the importance of proximal versus distal NVC of the facial nerve. Although some authors have suggested that NVC of the distal cisternal segment of the facial nerve near the porus acousticus (i.e. the peripherally myelinated portion of the nerve) may result in HFS,^31^^,^^32^ these observations are rare. Our study also confirms that arterial rather venous compression of the susceptible portion of the facial nerve should be the focus of preoperative MRI in patients with HFS. Patients in whom the offending vessel was intraoperatively found to be a vein have worse outcomes than those with arterial NVC.^33^ Our work provides evidence that these worse outcomes can be predicted pre-operatively. Furthermore, unlike patients with trigeminal neuralgia, increasing severity of NVC (i.e. contact versus deformation) is not a significant pre-operative predictor in patients with HFS. Our results in this regard are in agreement with other authors who compared the success rates in patients with severe NVC versus non-severe NVC found intra-operatively.^34^

The overall spasm-free status in this cohort of patients compares favourably with three large meta-analyses of MVD for HFS performed.^35–37^ But unlike the recent meta-analysis^35^ that found only initial versus repeat MVD to be a significant predictor of spasm freedom (and did not include imaging findings in their analysis), our work suggests that imaging, when tailored to evaluate for NVC, can provide statistically significant predictive information. Twenty years ago, Chung et al.^38^ evaluated 440 patients with HFS and commented that ‘there is a paucity of studies relating to evaluating the preoperative MR findings in terms of predicting the clinical outcome’.^39^ Their observation remains true 20 years later. While they did find a significant relationship between outcome and vascular indentation found on preoperative imaging or intra-operatively, the effect size was not described. Our work is the first paper, to our knowledge, that quantifies and compares the success rates for the different categories of imaging findings. Thus, the role of imaging is not merely to exclude other compressive masses, but to also report imaging findings that correlate with surgical outcome and better inform the pre-operative risk-benefit analysis and discussions with patients.

### Limitation of this study/analysis

There are several limitations to this study. This is a retrospective analysis, performed at a single centre with a dedicated cranial neuralgia program. The results may not be generalizable to all institutions. In addition, very few patients selected for MVD at our institution did not have arterial NVC of the susceptible portion of the facial nerve resulting in broad 95% CIs for several Odds Ratios.

## Conclusions

In conclusion, patients with arterial NVC of the susceptible portion of the facial nerve are much more likely to be spasm free following MVD than patients without this imaging finding. No statistically significant difference was seen when comparing the patients with arterial deformity versus arterial contact of the susceptible portion of the nerve; patients with both of these imaging findings pre-operatively are very likely to be spasm free following MVD. Patients with no NVC or venous only NVC are unlikely to be spasm free following MVD. These results provide valuable information for physicians counselling patients prior to MVD.

## Competing interests

The authors report no competing interests.

## Abbreviations

HFS =: hemifacial spasm
MVD =: microvascular decompression
NVC =: neurovascular compression
RDP =: root detachment point
RExP =: root exit point
SSFP =: steady-state free precession
T =: Tesla
